# Initial Stage of Nanoscale Imaging in Positive Tone Extreme UV Photoresists: The Influence of the Polymer Sequence

**DOI:** 10.1021/acsapm.5c03773

**Published:** 2025-12-19

**Authors:** Frances A. Houle, William Hinsberg, Jacob R. Milton, Qi Zhang, Cheng Wang, Samuel M. Blau

**Affiliations:** † Chemical Sciences and Molecular Biophysics and Integrated Bioimaging Divisions, 1666 Lawrence Berkeley National Laboratory, Berkeley, California 94720, United States; ‡ Columbia Hill Technical Consulting, Fremont, California 94539, United States; § Materials Science Division, Lawrence Berkeley National Laboratory, Berkeley, California 94720, United States; ∥ Advanced Light Source, Lawrence Berkeley National Laboratory, Berkeley, California 94720, United States; ⊥ Energy Storage & Distributed Resources Division, Lawrence Berkeley National Laboratory, Berkeley, California 94720, United States

**Keywords:** EUV photolithography, defined sequence polymers, poly(hydroxystyrene), poly(t-butyl methacrylate), radiolysis

## Abstract

Photolithographic patterning using extreme ultraviolet (EUV, 92.5 eV) light is a radiolytic process that initially forms electrons, radical cations, anions, and neutral radicals in the polymeric photoresist matrix. These species may participate in the chemical reactions that define the ultimate resolution of the printed image, and their concentrations and nanometer-scale stochastic variations in their formation influence printed image quality. Proposals have been made that polymer chain uniformity may be advantageous in reducing stochastics due to spatial inhomogeneities, and this aspect of radiolysis is examined in this work. We have simulated the initial subpicosecond stages of the imaging process for a series of photoresist films that are identical in composition but vary in their polymer chain structures. We use detailed, physically accurate stochastic reaction-diffusion calculations to evaluate the influence of defined sequence and random copolymer structures on radiolytic spur formation, i.e., a cluster of species formed by electron-polymer interactions that defines the initial spatial characteristic of the imaging process. Predictions of electron thermalization in the present work are shown to be consistent with the literature, indicating that our overall computational approach for ultrafast nanoscale processes is sound. The computational results show that the polymer sequence has no significant effect on the spur composition. This suggests that any potential imaging improvements to be gained by sequence control must originate from postimaging lithographic process steps.

## Graphical abstract

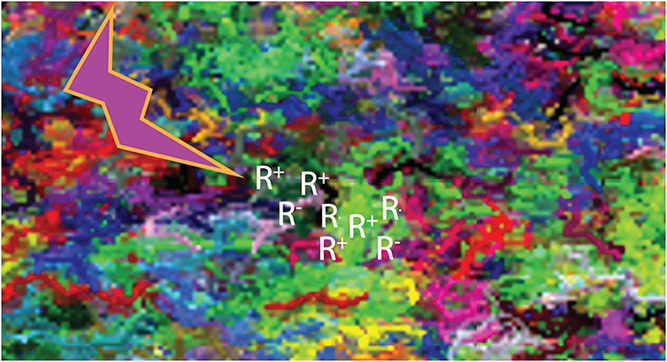

## Introduction

Lithographic patterning using extreme ultraviolet light (EUV, 13.5 nm, 92.5 eV) is used to produce the most advanced semiconductor devices because of its ability to form features at the few tens of nm half-pitch scale.^1,2^ The patterning process involves exposure of an approximately 30 nm thick photoresist film to EUV light, forming either positive tone or negative tone latent images that are subsequently thermally processed and developed. Positive tone photoresists for this wavelength have evolved from the Environmentally Stable Chemically Amplified Photoresist (ESCAP) class of photoresists developed for deep ultraviolet (DUV) lithography.^3^ These are complex mixtures of co- or terpolymers of random composition and finite polydispersity, mixed together with a DUV-sensitive photoacid generator (PAG) and a base quencher to control the imaging.^4^ The DUV wavelength currently in widest use for printing is 193 nm, where photoabsorption by the PAG results only in photochemical transformations in the resist film. EUV illumination, on the other hand, involves a complex sequence of ionization and excitation processes for image formation that have been elucidated in key investigations published since the early 2000s.^5–9^

The initial light absorption event, which is controlled by the excitation cross sections for atoms in the film,^10^ results in ejection of a high energy electron (about 80 eV) following ionization of the absorbing moiety. As illustrated in Figure 1, this electron initiates a short cascade of scattering events with about three additional ionizations, forming a radiolytic spur containing radical cations, anions, free radicals, and species in electronically excited states.^8^

**Figure 1:**
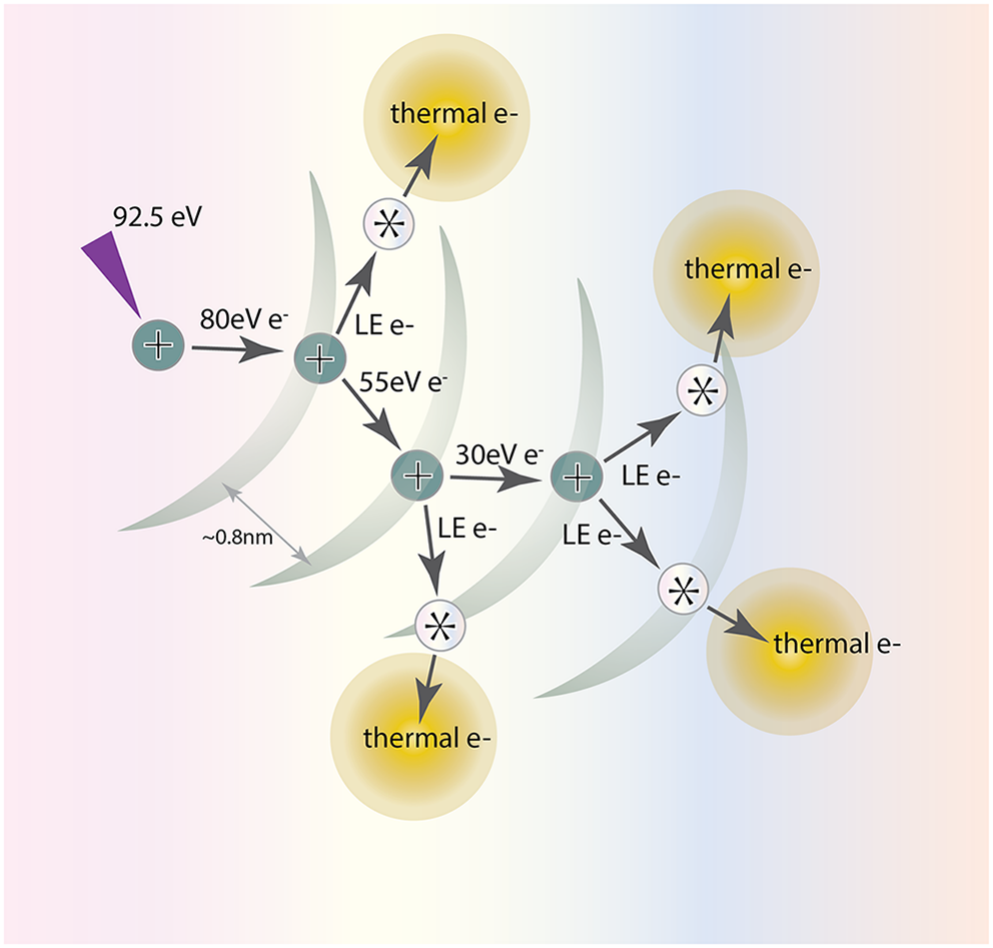
Schematic of spurs formed by EUV (92.5 eV) photon absorption in a polymer film. The mean free path of the electrons is about 0.8 nm. Each ionization event following the initial photoionization generates 2 electrons (including at least one low energy (LE, ∼5 eV) electron) which lose energy through further ionization or by exciting a resist component (*). Thermal electrons migrate freely in the film and are eventually trapped by neutral or cationic species.

Because of the high EUV photon energy and moderate dose required for patterning,^2^ photon absorption events are sparse in the resist film. For example, to print a 30 nm diameter via an interconnect feature in a photoresist film 30 nm thick that requires an imaging dose of 45 mJ/cm^2^, about 1740 photons are absorbed, corresponding to 1 per 124 nm^3^. Accordingly, each photon can be considered to interact with pristine resist material with rarely overlapping spurs.

The actual latent photolithographic image is not a perfect pattern because it is created by random photon absorption events in a randomly ordered multicomponent film, generating nanometer-scale statistical variations in the chemical state. This poses a challenge to control the molecular – level quality and uniformity of the printing process from even the initial moments. A number of strategies have been proposed to improve image quality by improving ordering and reducing inhomogeneities in the resist film, in particular, the use of molecular resist films and the use of defined sequence polymers with uniform length and structure.^2,11^

In a recent study, a positive tone photoresist using a defined sequence polypeptoid polymer platform has shown sequence-dependent imaging behavior in the final, developed resist image when exposed to DUV light.^12,13^ This is an interesting result given that the packing structure of a film prepared from a defined sequence polymer does not differ significantly from one formed from a polydisperse random copolymer.^14^ Because the polypeptoids are envisioned for EUV lithographic applications,^13^ how imaging processes in them compare to those in random copolymers at 13.5 nm exposure is a central question. In this study, we calculate the initial stage of spur formation in a set of five defined sequence and polydisperse copolymer films. All have the same positive tone ESCAP class photoresist composition, varying only the sequence of the monomers in the polymer chains. We use polymer packing simulations and stochastic reaction-diffusion modeling supported by experimental measurements and theory to examine the spatial and temporal aspects of EUV-induced ion and radical formation on the nanometer and femtosecond scales in these materials. The simulation results enable a detailed comparison of the image as a function of polymer chain structure to be made and show that any influence of the polymer sequence that might occur is not a result of initial radiolysis during exposure.

## Computational and Experimental Methods

The radiolysis physics is simulated using reaction-diffusion stochastic kinetics, a technique that models molecular-level events including randomness and fluctuations to predict observable phenomena. While this study is primarily computational, additional key supporting density functional theory (DFT) calculations and X-ray measurements are included to provide essential information for model construction.

### Composition of the Photoresist Used for the Modeling

The photoresist investigated here is shown in Scheme 1. It is a copolymer of 4-hydroxystyrene (PHS) and *tert*-butyl methacrylate (TBMA) in a 40:60 ratio. A photoacid generator (PAG), which generates protons for image formation, and a base quencher (quencher), which moderates the proton populations spatially, are included in the formulation. Utilizing the film density (see below), this composition results in concentrations of 2.52 and 3.78 M of the copolymer components and 0.297 and 0.187 M for the PAG and quencher, respectively. The PHS and TBMA in the copolymer are treated as separate species when they undergo photophysical events. Additionally, although the PAG and quencher salts are associated ion pairs within the film, the triphenyl sulfonium (TPS) cation and the anions are treated as independent species in the exposure mechanism. For comparison to the existing literature, a set of simulations using the same reaction scheme are also performed for pure PHS, whose concentration of 9.65 M is determined from its bulk density of 1.16 g/cm^3^ (vendor Web site). The thin film has been determined to have the same density.^15^

**Scheme 1:**
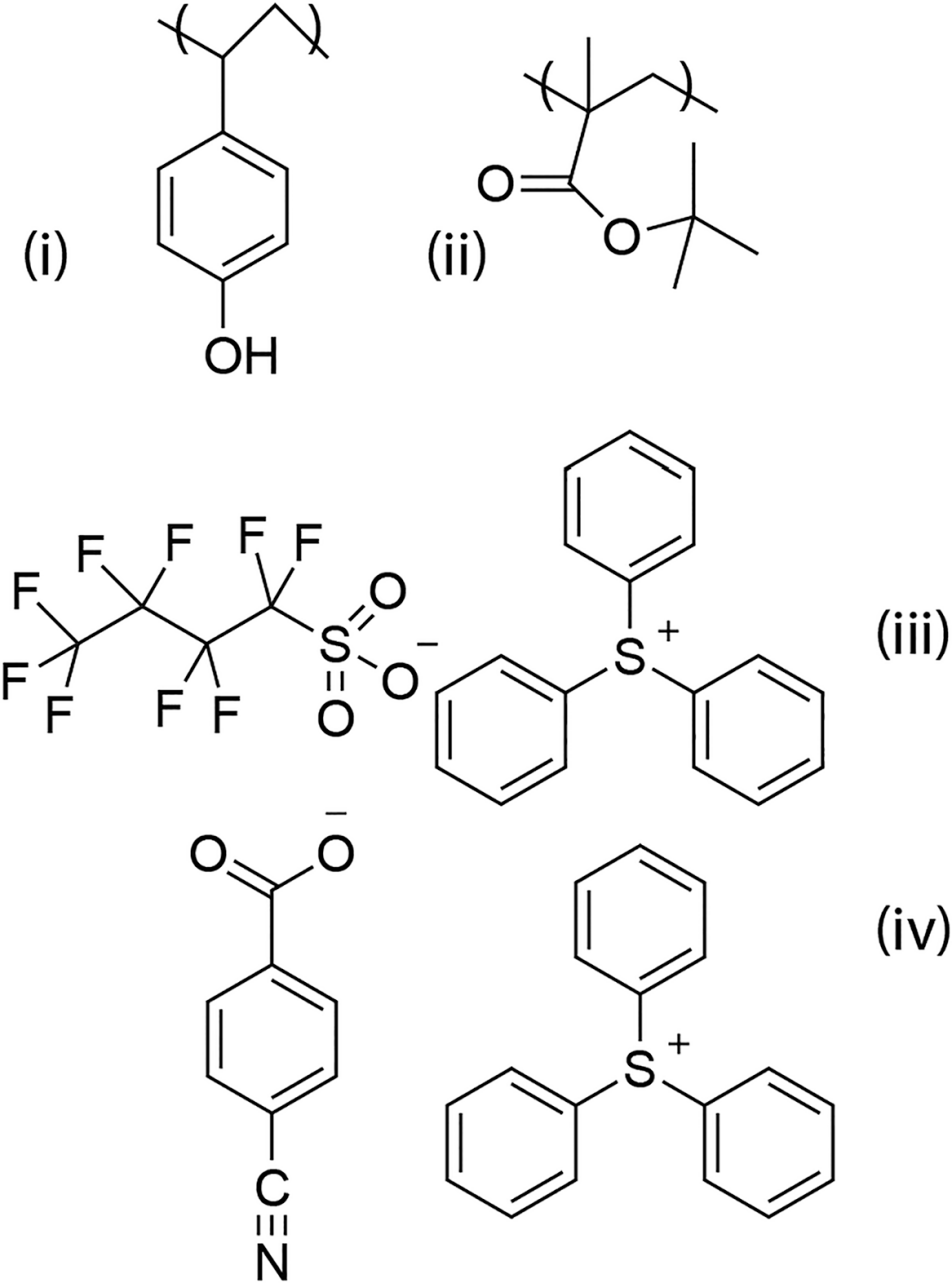
Photoresist Components used in This Work. (i) 4-hydroxystyrene (PHS), (ii) Tert-butyl Methacrylate (TBMA), (iii) Perfluorobutane Sulfonate (PAG) Triphenyl Sulfonium (TPS), (iv) 4-cyanobenzoate (Quencher) Triphenyl Sulfonium (TPS). The Polymer Composition is 40% (i) and 60% (ii). (iii) and (iv) are 20 Weight% and 10 Weight%, Respectively

### Polymer Film Construction

The polymer packing code package Polyscope is used to establish the spatial characteristics of the film compositions for the reaction-diffusion kinetics simulations.^14,16^ As shown in Figure 2, five variations of a 30-mer 40:60 polymer structure are modeled: a monodisperse random chain, a random chain with a polydispersity of 1.5, a defined sequence chain with alternating TBMA and PHS, a defined sequence chain with five 6-member blocks, alternating between TBMA and PHS, and a defined sequence chain with two blocks of 16 TBMA and 12 PHS. The construction process involves random placement of 90,000 monomers into approximately 3000 chains to form a film that has the photoresist film density (described below) and insertion of PAG and quencher molecules into free space elements to create the final photoresist mixture. Each film is 25.6 nm thick (y direction), 31 nm long (x direction), and 26.5 nm wide (z direction). Further details are provided in the Supporting Information (SI) Section S1. All films have the same dimensions and visibly similar or dissimilar spatial distributions. Calculations of radial distribution functions can provide additional detail on these distributions^14^ but were not performed for the present work.

**Figure 2:**
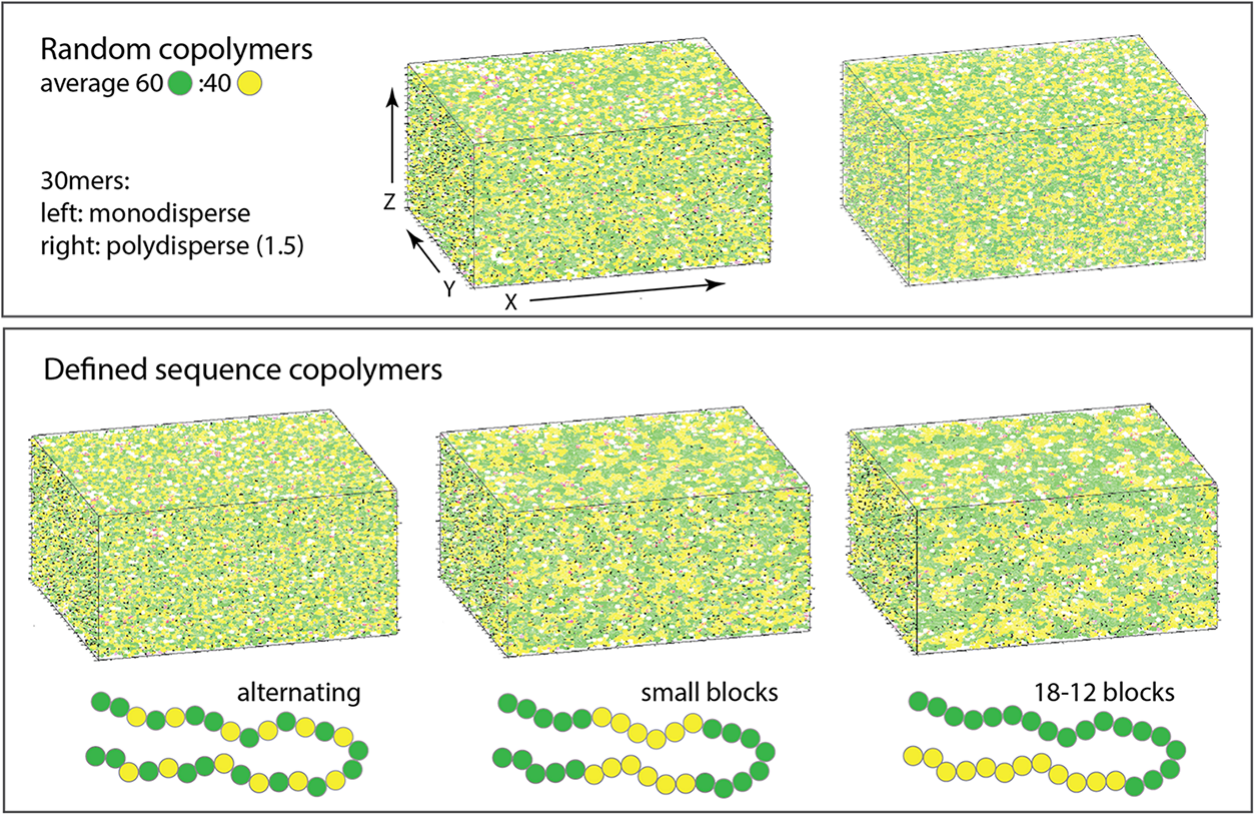
Five polymer assemblies examined in this study. The packing code arranges the chains into 31 × 25.6 × 25.6 nm^3^ film blocks, with TBMA (green) at 60%, PHS (yellow) at 40%, and the PAG and quencher added in (pink and dark red, respectively).

For comparison to prior literature computational studies, a pure PHS film is also examined. No polymer packing calculations are necessary for single component materials.

### Reaction-Diffusion Simulation Methodology

Radiolysis simulations are performed using Kinetiscope, an open access stochastic reaction-diffusion simulation package.^17^ Inputs consist of initial concentrations determined by Polyscope calculations and formulation data. The spatial distribution is incorporated into a three-dimensional (3-D) grid that includes a reaction mechanism and diffusion steps (SI Section S2). The simulator represents molecules with particles proportion to their initial concentrations. In the present work, one particle represents 1.66 × 10^–24^ moles of each species present, thus enabling events to be tracked at the level of molecules and electrons throughout the system. Kinetiscope propagates a simulation by randomly selecting among probability-weighted reaction and diffusion events throughout the 3-D simulation volume, where the probability is calculated using the instantaneous rate laws for each step in the mechanism.^18^ It produces a physically accurate time base when accurate concentrations and primary rate coefficients are used. Because the method is governed by Poisson statistics, the reactant and product distributions are analyzed to obtain mean and standard deviation values for the amounts of each species in the combined 64-spur set as a function of space and time. This computational method allows use of auxiliary marker species, as detailed in SI Section S2, which enables a more comprehensive analysis of the simulation results.

### Physical Model Construction

In this study, reaction-diffusion processes within a photoresist film are simulated using a one-dimensional (1-D) model framework. The films in Figure 2 are segmented into 3968 3.21 nm (y dimension) × 3.21 nm (z dimension) × 0.5 nm (x dimension) compartments, each with a volume of 5.15 nm^3^ containing about 24 molecular moieties. A linear array of 62 compartments along the x direction represents the volume in which a spur can be formed. The compartment size was chosen as the smallest in which all resist components could be present, given the photoresist composition. Due to the randomness of the packed film, generally no compartment has the exact nominal composition, however. The compartment and 1-D array sizes correspond to natural radiolytic distances, as determined in the literature. Modeling has shown that the Onsager length for electrons recombination is about 15 nm in polystyrene,^19^ which has a density in the same range as the resist polymer modeled here, so the total 1-D framework length was chosen to be about double that value to ensure that there are negligible boundary effects. The electron scattering distances for high energy electrons in poly­(methylmethacrylate) is 0.79 nm,^20^ up to two 0.5 nm thick compartments, and the electron deexcitation distance from ∼5 eV to thermal energy in poly­(hydroxystyrene) is about 3 nm, corresponding to six compartments.^9^ The compartment y and z dimensions thus accommodate some amount of potentially off-angle electron motion without leaving the 1-D assembly.

The chemical composition of all compartments is read into a system of 64 1-D arrays of compartments, where each array is interconnected along the *x*-axis using diffusion paths. These paths allow electron motion within a 1D array, while chemical moieties remain fixed in space. Adjacent 1-D arrays are fully independent (no diffusion in the y and z directions).

One photon is absorbed in the first compartment of each array, initiating the spur formation process at the beginning of the reaction-diffusion simulations. By simulation of 64 separate spurs in one calculation, 64 independent reaction-diffusion histories are obtained on a common time base. This enables event statistics to be determined as a function of location along the 1-D array and time from the simulation results.

### Radiolysis Model

The EUV photophysics determine the structure of the radiolysis model, whose processes are informed by prior studies.^6–8^ The quantum yield of primary and secondary cation formation events has been determined to be about 4.^8^ Modeling has determined that for PHS, the electron energy lost per collision is 22.2 eV.^21^ With the initial photoelectron at 80 eV,^22^ the remaining electron ionization steps are assumed to involve a loss of 25 eV, resulting in 55, 30, and 5 eV scattered electrons.

The general primary steps are the same for all five resist components, denoted as C (polymer moieties, PAG and quencher anions, and TPS cation)

1 $$C+\mathrm{h\nu}\to C^{+}+{\mathrm{electron}}_{80\mathrm{ev}}$$

2 $$C+{\mathrm{electron}}_{80\mathrm{eV}}\to C^{+}+{\mathrm{electron}}_{55\mathrm{eV}}+\mathrm{LEE}$$

3 $$C+{\mathrm{electron}}_{55\mathrm{eV}}\to C++{\mathrm{electron}}_{30\mathrm{eV}}+\mathrm{LEE}$$

4 $$C+{\mathrm{electron}}_{30\mathrm{eV}}\to C^{+}+2\mathrm{LEE}$$

5 $$C+\mathrm{LEE}\to C^{*}+\mathrm{TE}$$

6 $$\mathrm{TE}+C^{+}\to C$$

7 $$\mathrm{TE}+C\to C^{‐}$$

8 $$C^{*}\to C$$

where LEE is a ∼5 eV low energy electron that cannot ionize a neutral species, and TE is a thermal electron, 0.1 eV. The detailed scheme for all species is presented in SI section S2. It should be noted that light absorption in the first compartment and electron interactions in all compartments can take place with any species, so event sequences evolve randomly.

Rate coefficients for the scheme are described in SI Table S2 and briefly summarized here. The coefficient for step (1) is calculated from the 13.5 nm absorption coefficients for each component C, which are determined by the atomic absorption cross sections (SI Tables S3 and S4).^10^ Rate coefficients for electron ionization and relaxation (steps (2) – (5)) are estimated from the time an electron of that energy takes to travel the mean inelastic scattering distance in the material, 0.79 nm.^20^ Rate coefficients for recombination of TE with radical cations (step (6)) or trapping of TE by neutrals to form anions (step(7)) are assumed to be diffusion-controlled and are estimated from the Smoluchowski and reduced Debye equations.^23^ Relaxation of excited states is assumed to occur after intersystem crossing to the triplet manifolds, consistent with observations that significant quantities of triplet species are formed in electron radiolysis experiments.^24^ A typical phosphorescence lifetime of 0.5–1 ms is used to estimate the rate coefficient for step (8).^25^ Diffusion coefficients (Table S5) are calculated from the time required for an electron of a specified energy to travel 1 nm.

### Theoretical Estimates of Ion Formation Energetics

Both positive and negative ions are formed during the radiolysis cascade. DFT calculations are performed to determine which electron detachment and attachment events are energetically accessible. SI Section S3 provides details of the methods used and the estimated values. From them, electron detachment from TPS, which is positively charged, and electron trapping by neutral PHS can occur and are included in the reaction scheme (SI Tables S2 and S6). To attach a thermal electron, a moiety must have a vertical electron affinity which does not preclude attachment (i.e., is not substantially below zero), have a positive adiabatic electron affinity (i.e., is thermodynamically favorable after structure relaxation), and not be initially negatively charged, in which case electrostatic repulsion would most likely prevent attachment. These chemically intuitive rules thus indicate that radical cations, TPS^+^, and PHS are the only species that can undergo thermal electron attachment in the system.

### Measurement of Resist Film Density

The polymer packing and reaction-diffusion simulations both require knowledge of the resist density to ensure an accurate spatial setup. The polymer packing calculation utilizes the distance between monomer units as an input. The density of the resist formulation is required for this distance, but it has not been reported. Because very thin polystyrene and poly­(methylmethacrylate) film densities can deviate significantly from estimates from bulk material densities,^26^ this quantity has been measured for the present study. The method utilizes an index of refraction measurement performed at Advanced Light Source (ALS) beamline 6.3.2 at Lawrence Berkeley National Laboratory. Details are provided in SI section S4. The value obtained for a 38 nm thick film of the polymer (no acid generator or base quencher components) is 1.075 ± 0.05 g/cm^3^. Utilizing data from the Web sites of the chemical vendors of each component, the fully formulated resist is estimated to have a density of 1.08 g/cm^3^, in good agreement with the measurement. The less precise value has been used in the packing and reaction-diffusion simulations because of the approximations made in model construction. At this density, approximately 25 molecular moieties are present in each compartment.

## Results and Discussion

### EUV Radiolysis of Pure PHS

Much of what is known about radiolysis of photoresist materials has been obtained from pure homopolymer studies or mixtures of a homopolymer with a PAG to understand the acid generation mechanism during exposure.^8,22,27^ Excitation can be by electron beams or EUV photons; however, the two do not produce identical product distributions.^19^ Our simulation results for spatial distributions of the products formed when all radiolysis processes have ended are shown in Figure 3. This time is approximately 1 ms, determined by the slow decay of excited states assumed in this work. Because the excited states are mostly relaxed at the end of the simulation, the total number of formation events is more useful to assess the importance of excitation vs ionization. Plots as a function of time show that although significant quantities of thermal electrons are generated during the course of the simulation, all are trapped (forming anions) or have recombined with PHS^+^ in less than a ps (SI section S5 and Figure S2,) so there are no solvated electrons at the end of the simulation. It is evident that radiolytic product formation extends well outside of the initial absorption volume, which is 0.5 nm wide.

**Figure 3:**
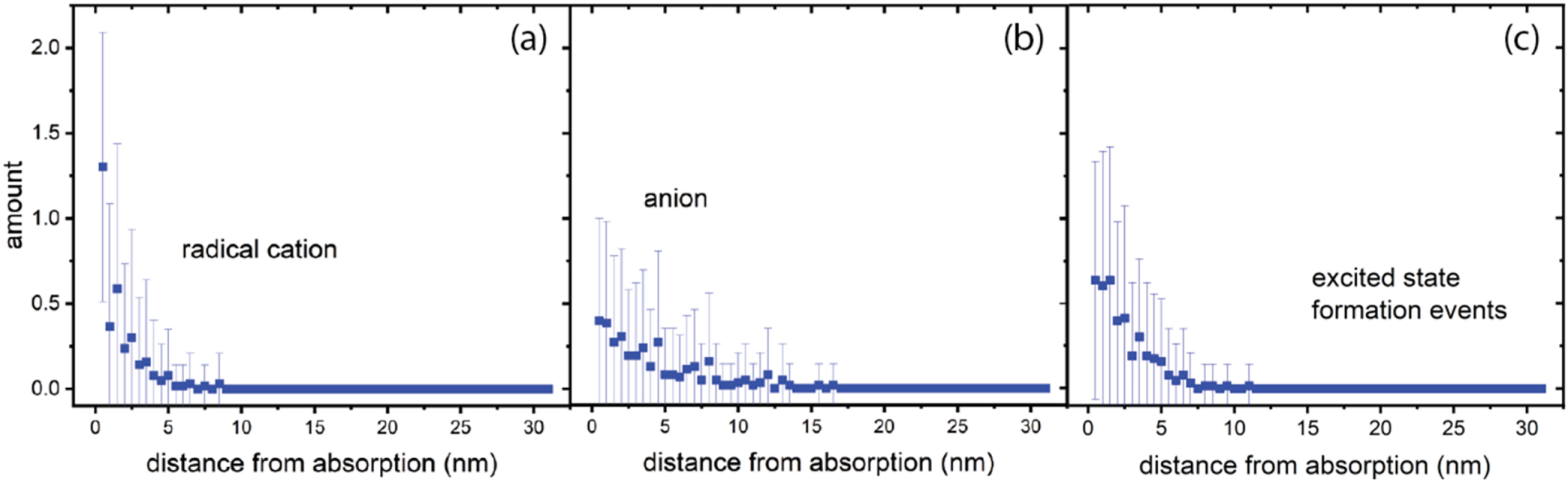
Terminal spatial distribution of primary radiolysis products in PHS, numbers per 5.1 nm^3^ compartment as a function of distance from the first compartment where EUV absorption occurs. Points are mean values, vertical bars are the standard deviation for the set of 64 separate 1D volumes. (a) PHS radical cations, (b) PHS anions, (c) location of excited state formation events occurring throughout the simulation.

Our predictions of how EUV light absorption results in ionization in the photoresist can be compared to prior modeling work from the literature that has focused on homopolymers (polystyrene and polyhydroxystyrene, neat and with inclusion of photoacid generators). Simulations of thermalized electron distributions following EUV absorption have shown that the radical cations formed extend about 4 nm from the absorption point, and thermalized electrons to about 8 nm.^28^ The simulation results in Figures 3 and S3 are consistent with these findings. PHS radical cations are found within 5 nm of the absorption location, with a 1/*e* ^2^ distance of 4.3 nm determined by fitting the data points to an exponential function. Thermal electron spatial distributions evolve continuously throughout the simulation, with none remaining at the end, so PHS anion formation via step (7) is taken to represent their extent of diffusion. The 1/*e* ^2^ value for this distance is 6.5 nm, with a maximum extent of about 10 nm, in agreement with that of the earlier study. Additional computational studies of radiolysis in PHS under EUV excitation provide estimates of the electron thermalization distance, which is the excited state formation distance in the present study, of about 4 nm^29^ and 3.2 nm.^9^ The 1/*e* ^2^ distance is 3.8 nm in Figure 3, in good agreement with the literature values. The general consistency between the results of the computational approach used here and prior work supports the use of our methods to predict radiolysis in typical organic photoresists.

### EUV Radiolysis of a Fully Formulated Photoresist

In this work, the structures examined are individual spurs resulting from the absorption of a single photon in the photoresist film. Figure 4 shows the mean compositions and standard deviations of 64 separate 1-D arrays prior to light exposure obtained from the packed films in Figure 2. The values shown are obtained for each ∼5 nm^3^ compartment in the 1D array. Numerical values for each plot are included in the SI (data spreadsheet) to provide quantitative information about statistical variations. Figure 4 shows that the initial compositions as a function of location are indistinguishable between the 5 polymer chain variations; however, the standard deviations vary to a small degree, especially across the three defined sequence cases. These differences are also qualitatively visible in Figure 2.

**Figure 4:**
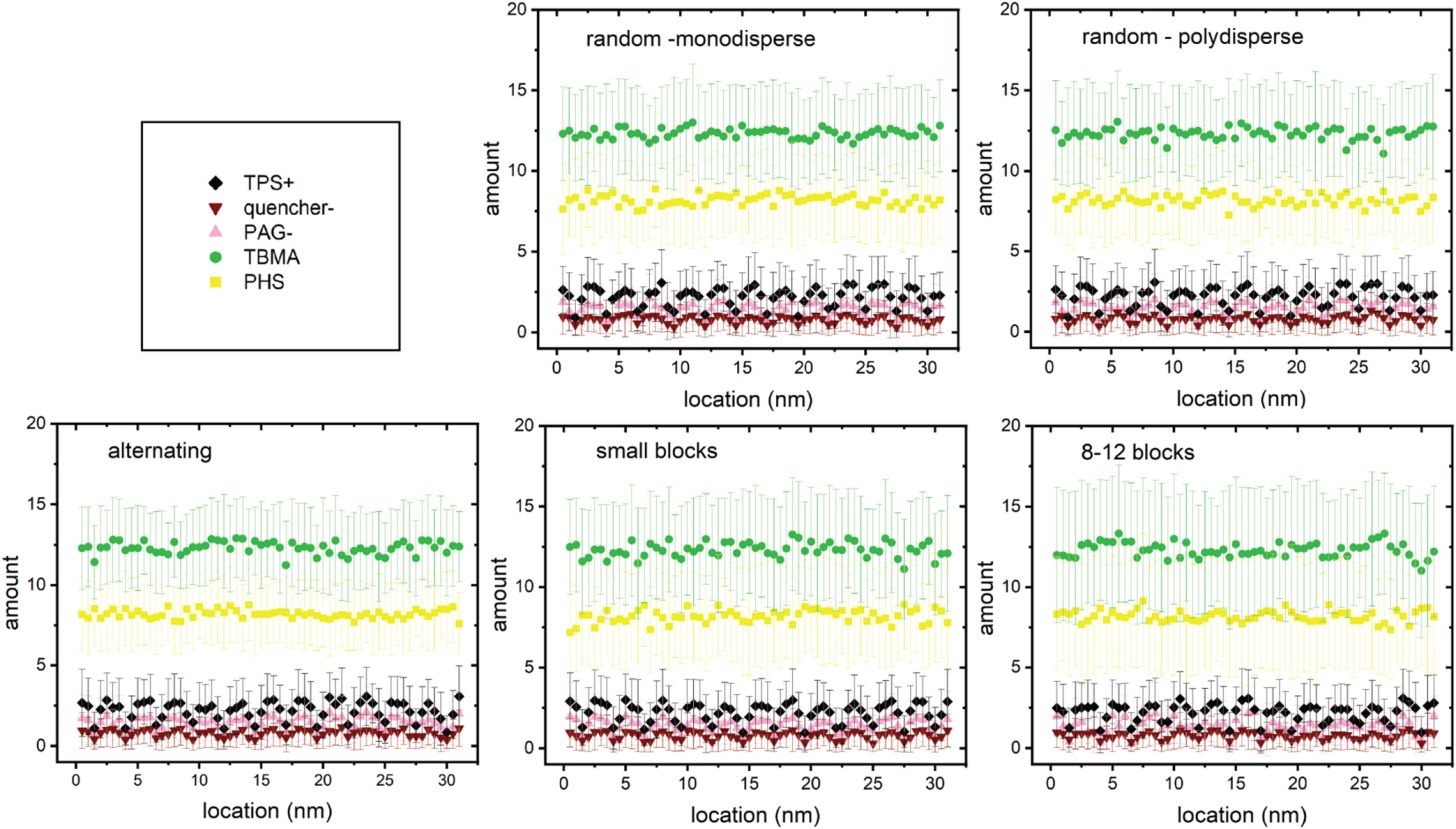
Spatial distributions of resist components for five variations in polymer structure (definitions in Figure 2). The data presented are the mean compositions of 64 individual 62-compartment 1-D arrays, and their standard deviations. The apparent periodicity of the mean values of the minor components is likely to be due to the computational algorithms used.

Photoabsorption generates highly energetic electrons that rapidly lose energy down to the range of ∼5 eV, forming an LEE that does not participate in ionizing collisions. The time scale for this process is about 10 fs, as shown in Figure 5, and at any one time there is on average 0.5 LEEs present within the spur volume. The LEEs have relatively low abundance since they lose energy rapidly by electronically exciting molecular components in the photoresist. The thermal electrons (TE) formed are much more numerous, reaching a peak number of about 2.5 within the spur volume at 10 fs and disappearing completely by 0.1 ps as a result of recombination with radical cations or TPS cations or being trapped by PHS to form radical anions.

**Figure 5:**
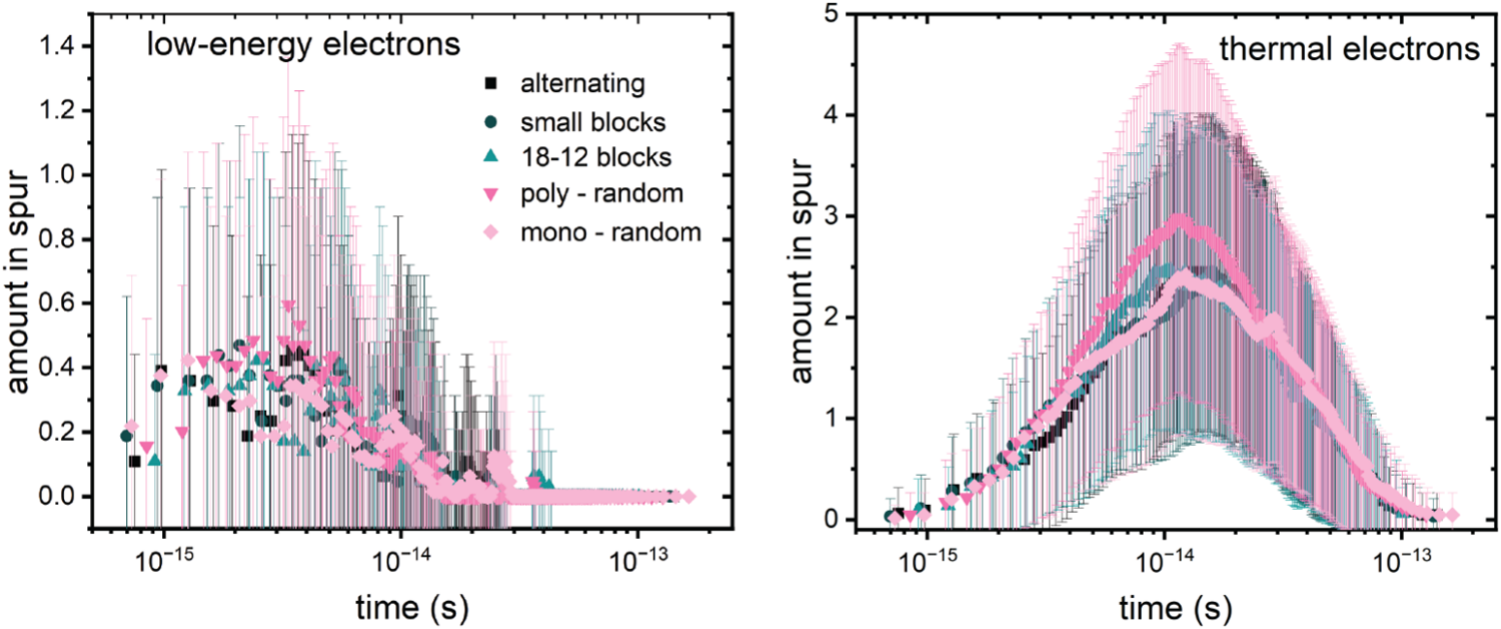
Total electron populations in each spur volume (62 × 5.15 nm^3^ = 319.3 nm^3^) as a function of time after photon absorption by any of the photoresist components. Electrons are completely consumed at about 0.1 ps.

The time histories of each species are listed in Figure 6. Because the present calculations focus entirely on initial radiolysis processes within 10–100 fs, the excited states decay rather than undergoing energetically accessible chemical reactions. SI Figure S4 shows the spatial location of ionization and decay processes, revealing that they largely overlap in space, out to about 5 nm from the initial EUV excitation event.

**Figure 6:**
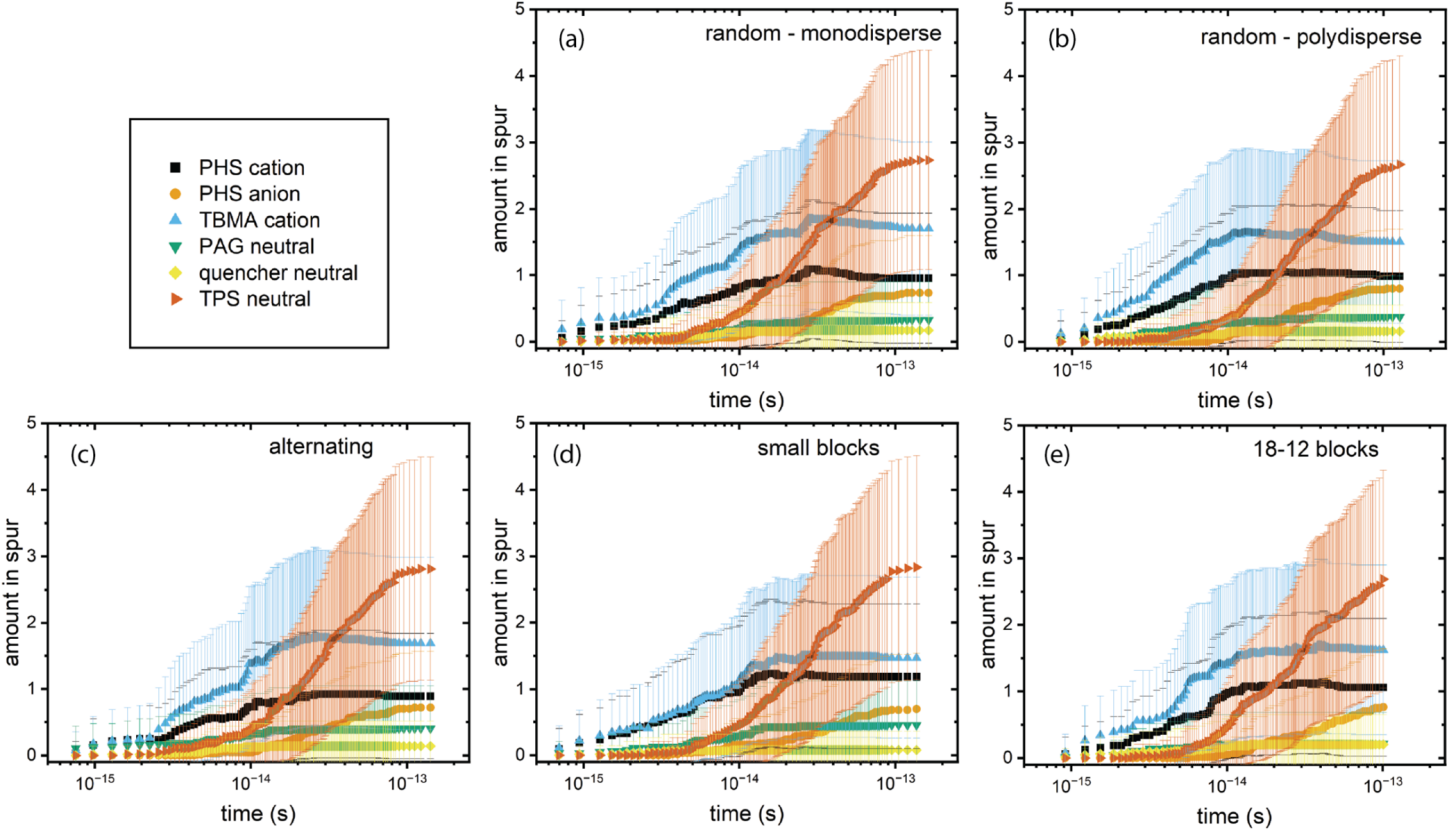
Time-dependent formation of primary radiolysis products (a–e). These are totals in each 319.3 nm^3^ spur volume.

On average, the simulation results predict that the total number of primary initial radiolysis products in each spur are 3 radical cations, 3 neutral radicals, and 0.7–0.8 radical anions per photon absorbed for all resist polymer structures (SI Table S7). These are small populations. They can be expected to react subsequently with any products formed from excited states as well as other nearby resist components in the films. How these reactions might influence developable image quality resulting from EUV exposure compared to DUV exposure which only produces acid is currently under investigation.

The first products to be formed are a mixture of primarily radical cations from the polymer moieties, doubly ionized triphenyl sulfonium, and neutral radicals formed from the PAG and quencher anions. This process is largely complete in about 10 fs. After this point, TE capture by PHS and triphenyl sulfonium generates PHS radical anions and triphenyl sulfide neutral radicals. The spatial distributions of the radiolysis products are shown in Figure 7.

**Figure 7:**
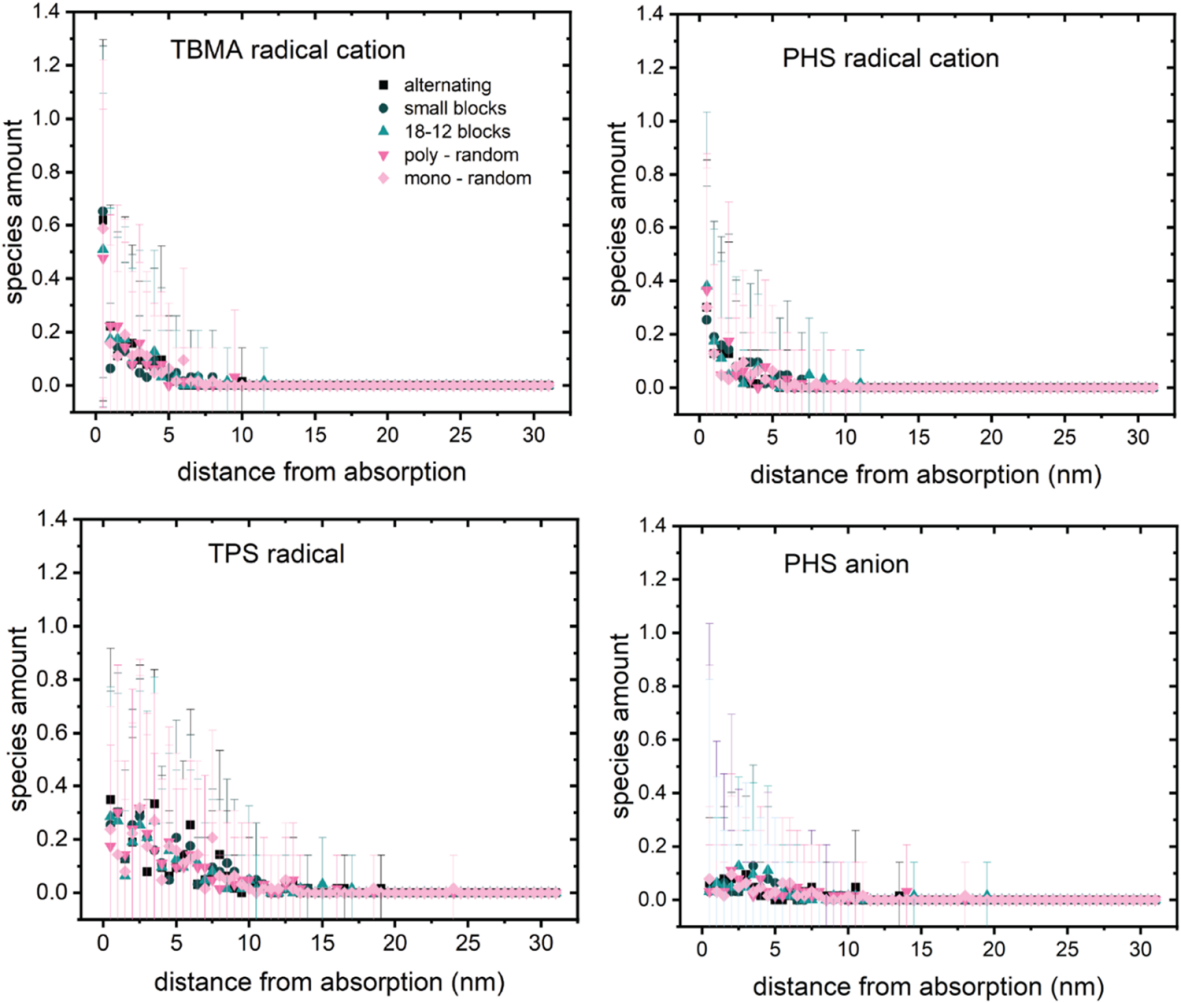
Spatial distributions of radiolysis products formed. Amounts are in each 5.15 nm^3^ compartment in the 62-compartment 1D simulation volume.

Monte Carlo simulations of electron energy losses in a photoresist composition similar to the one investigated in this work indicated that the most probable thermal electron travel distance is 3–5 nm, with a distribution extending to 8–10 nm.^7^ Figure 7 shows that anions and radicals, formed by trapping thermal electrons, are present in this range. These distances are comparable to those predicted for PHS in this work, indicating that the higher density of PHS (1.18 g/cm^3^)^15^ does not significantly affect the overall radiolysis process. Accordingly, the results of the present simulations are in agreement with prior work in the literature and can be used to assess the influence of the polymer film structure.

The data in Figures 5–7 show that the outcomes of the initial processes launched by EUV photoexcitation are insensitive to the details of how individual polymer chains are constructed. Even though Figure 2 shows clearly that the polymer chain structure can lead to inhomogeneities and that there is significant spatial variability in film composition, these inhomogeneities do not lead to notable variability in product distributions or in lifetimes when averaged over the number of individual instances examined in this work. Returning to the 30 nm via example given in the introduction, if a 45 mJ/cm^2^ dose is required to expose the resist, 1740 EUV photoabsorption events are required. If the influence of polymer spatial distributions is not discernible for a collection of 64 spurs, they will be even less so when such a large number of events accumulates to form a nanoscale image. Therefore, specifically targeting the polymer structure to reduce stochastic variations is not likely to improve the uniformity of the latent image at the initial exposure stage.

## Conclusions

This study is designed to evaluate the influence of polymer structure on reactive intermediate formation during the first moments of radiolysis in positive tone organic photoresist systems. By integration of detailed simulations of the initial spatial distribution of resist components, including statistical variations, with stochastic reaction-diffusion kinetics for the ionization, excitation, and electron trapping reactions, a nanoscale picture of image formation by EUV light absorption has been developed.

The simulation results described here are compared, where possible, to prior results in the literature. The electron thermalization distance has been a particular focus, and the good agreement that results from the present combination of polymer packing and stochastic reaction-diffusion simulations is evidence that the computational approach is a useful way to examine details of the imaging process that are not readily accessible experimentally.

The time histories and spatial distributions of final radiolysis products are readily compared for all five polymer structures, and it is clear that whether the polymer chain is random or a defined sequence has no significant influence on the spatial characteristics of the image that is formed. Direct simulations of the full chemistry resulting from radiolysis at ambient temperature in vacuum and of the postexposure bake process in air are required to establish whether polymer structure influences the final developable image. Beyond addressing this particular question, calculations that include both radiolysis and full chemistry can more generally be used to calculate latent images and developable images for a range of photoresist formulations. Comparison of these predictions to results from in situ resonant soft X-ray scattering measurements of patterned samples would be a particularly useful means of both validating the simulations and learning more about the pattern formation process itself.

## Supplementary Material



## Notes

Parameters for polyscope simulations; radiolysis reaction-diffusion kinetic model including reaction steps, rate coefficients, and diffusion coefficients; theoretical estimates of electron attachment and detachment energies; measurement of photoresist film density using X-ray reflectivity; additional figures showing thermal electron time history in PHS, fits to determine electron transport distances and location of ionization and excited state formation events in fully formulated photoresist; table of radiolysis products remaining in the spur volumes; and data tables used in figures (Figure data - spur simulations 2025 Houle et al.xlsx) (PDF)
