# Supplementary material for: Initial Stage of Nanoscale Imaging in Positive Tone Extreme UV Photoresists: The Influence of the Polymer Sequence
Source: ACS Appl Polym Mater. 2025 Dec 19;8(1):361–9. doi: 10.1021/acsapm.5c03773 (PMC12797188; doi:10.1021/acsapm.5c03773)
Supplement: Supplementary file 1 [file ap5c03773_si_001.pdf]

Supporting Information

Initial stage of nanoscale imaging in positive-tone extreme UV photoresists: the influence of polymer sequence

Frances A. Houle [1], William Hinsberg [2], Jacob R. Milton [3], Qi Zhang [3], Cheng Wang [4], Samuel M. Blau [5]*

[1] Chemical Sciences Division, Lawrence Berkeley National Laboratory, Berkeley, CA 94720
[2] Columbia Hill Technical Consulting, Fremont, CA 94539
[3] Materials Science Division, Lawrence Berkeley National Laboratory, Berkeley, CA 94720
[4] Advanced Light Source, Lawrence Berkeley National Laboratory, Berkeley, CA 94720
[5] Energy Storage & Distributed Resources Division, Lawrence Berkeley National Laboratory, Berkeley, CA 94720

* Email: fahoule@lbl.gov

Table of Contents:

Section S1. Polyscope parameters for film structure calculations

Section S2. Radiolysis model

Section S3. Theoretical estimates of ionization energies

Section S4. Density measurement by X-ray reflectivity

Section S5. Additional simulation results

References

Section S1. Polyscope parameters for film structure calculations

The polymer packing code Polyscope[1] has been used to create the chain and monomer distributions for the kinetics simulations. 90000 monomers are assembled into chains in a film slab 31 x 25.6 x 25.6 nm. The top and bottom surfaces are planar and the 4 sides are assumed to be connected using period boundary conditions. The input parameters are calculated from the resist film density of 1.08 g/cc or 4.392 monomers per nm^3 as described in the main paper and in Section S4 below. The monomer radius is set to 0.305 nm, the bond length is 0.55 nm, and the bond angle is 70.5°. All polymer chain distribution calculations have the same random number seed(s). The resulting monomer distributions are shown in Figure 2 in the main paper. A corresponding chain distribution is shown in Figure S1. After the packing is complete, the film slab is divided into a set of 62 1D strips of 3.21 x 3.21 x 0.5 nm compartments for the kinetics simulations, each compartment containing the monomers present in the chain fragments within it. Monomers split across compartment boundaries during the division process are placed into one compartment based on where their fraction is greatest.

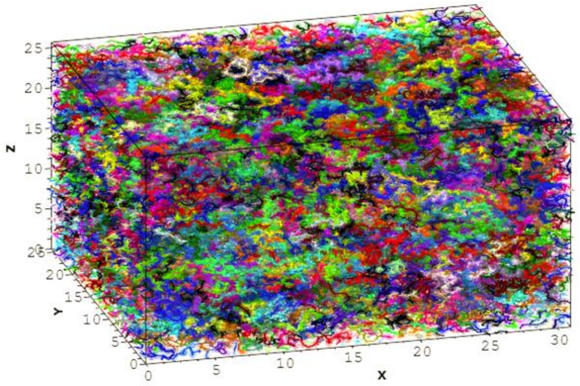

Figure S1. Typical polymer chain distribution created by the polymer packing code. Each chain in the slab has the prescribed sequence for the case under study (defined or random).

Section S2. Radiolysis model

The radiolysis model defines the processes occurring for each of the 5 species in the formulation. The underlying assumption is that the two polymer moieties, the PAG and quencher anions and the triphenylsulfonium cation interact independently with light and with electrons even though they are in close proximity on the polymer chain or in the PAG and quencher salts. The notation used for the photoresist components and mechanism is shown in Table S1. The mechanism is shown in Table S2, and details of rate coefficient calculations are in Tables S3 and S4.

Table S1. Species used in the radiolysis mechanism.

Name	Type	Definition, notes

quencher-	molecule	4-cyanobenzoate anion, related species are quencher0, a neutral radical, and excited states *
PAG-	molecule	perfluoro butane sulfonate, related species are PAG0, a neutral radical, and excited states *
PHS	molecule	4-hydroxystyrene, related species are PHS+ cation, PHS- anion, and excited states *
TBMA	molecule	*tert*-butyl methacrylate, related species are TBMA+ cation and excited states *
TPS+	molecule	Triphenyl sulfonium cation, related species are TPS0 neutral radical, doubly ionized TPS2+, and excited states *
excite	photon	single EUV photon, present only in the first compartment of the 1D array
eV_80	electron	kinetic energy of 80eV
eV_55	electron	kinetic energy of 55eV
eV_30	electron	kinetic energy of 30eV
LEE	electron	kinetic energy of ~5eV
TE	electron	kinetic energy of 0.1eV
quencher-_absorb	marker	quencher has absorbed EUV photon
PAG-_absorb	marker	PAG has absorbed EUV photon
PHS_absorb	marker	PHS has absorbed EUV photon
TBMA_absorb	marker	TBMA has absorbed EUV photon
TPS+_absorb	marker	TPS has absorbed EUV photon
ionization80	marker	80eV electron impact event
ionization55	marker	55eV electron impact event
ionization30	marker	55eV electron impact event
TEformation	marker	thermalized electron formation event
TErecombination	marker	thermalized electron-cation recombination
TEattachment	marker	thermalized electron trapping to form an anion
esdecay	marker	unimolecular excited state decay

Table S2. Mechanism for radiolysis of photoresist composition used in this work

Mechanism step	Rate coefficient
quencher- + excite => quencher0 + eV_80 + quencher_absorb	5.96×10^{12} s^{-1} [a]
eV_80 + quencher- => quencher0 + ionization80 + eV_55 + LEE	1.40×10^{15} L/mole-s [b]

eV_55 + quencher- => quencher0 + ionization55 + eV_30 + LEE	1.20 x10^{15} L/mole-s [b]
eV_30 + quencher- => quencher0 + ionization30 + 2 LEE	8.50 x10^{14} L/mole-s [b]
quencher- + LEE => quencher-* + TE + TEformation	1.70 x10^{14} L/mole-s [b]
quencher-* => quencher- + esdecay	2x10^{6} s^{-1} [c]
quencher0 + LEE => quencher0* + TE + TEformation	1.70 x10^{14} L/mole-s [b]
quencher0* => quencher0 + esdecay	2x10^{6} s^{-1} [c]
TE + quencher0 => quencher- + TEattachment	3.10x10^{12} [d]
PAG- + excite => PAG0 + ionization + eV_80 + PAG_absorb	2.27 x10^{13} s^{-1} [a]
eV_80 + PAG- => PAG0 + ionization80 + eV_55 + LEE	1.40x10^{15} L/mole-s [b]
eV_55 + PAG- => PAG0 + ionization55 + eV_30 + LEE	1.20 x10^{15} L/mole-s [b]
eV_30 + PAG- => PAG0 + ionization30 + 2 LEE	8.50 x10^{14} L/mole-s [b]
PAG- + LEE => PAG-* + TE + TEformation	1.70 x10^{14} L/mole-s [b]
PAG-* => PAG- + esdecay	2x10^{6} s^{-1} [c]
PAG0 + LEE => PAG0* + TE + TEformation	1.70 x10^{14} L/mole-s [b]
PAG0* => PAG0 + esdecay	2x10^{6} s^{-1} [c]
TE + PAG0 => PAG- + TEattachment	3.10x10^{12} [d]
PHS + excite => PHS+ + eV_80 + PHS_absorb	4.08 x10^{12} s^{-1} [a]
eV_80 + PHS => PHS+ + ionization80 + eV_55 + LEE	1.40x10^{15} L/mole-s [b]
eV_55 + PHS => PHS+ + ionization55 + eV_30 + LEE	1.20 x10^{15} L/mole-s [b]
eV_30 + PHS => PHS+ + ionization30 + 2 LEE	8.50 x10^{14} L/mole-s [b]
PHS+ + LEE => PHS+* + TE + TEformation	1.70 x10^{14} L/mole-s [b]
PHS+* => PHS+ + esdecay	2x10^{6} s^{-1} [c]
PHS + LEE => PHS* + TE + TEformation	1.70 x10^{14} L/mole-s [b]
PHS* => PHS + esdecay	2x10^{6} s^{-1} [c]
TE + PHS+ => PHS + TErecombination	4.1x10^{13} L/mole-s [e]
TE + PHS => PHS- + TEattachment	3.10x10^{12} [d]
TBMA + excite => TBMA+ + eV_80 + TBMA_absorb	5.40 x10^{12} s^{-1} [a]
eV_80 + TBMA => TBMA+ + ionization80 + eV_55 + LEE	1.40x10^{15} L/mole-s [b]
eV_55 + TBMA => TBMA+ + ionization55 + eV_30 + LEE	1.20 x10^{15} L/mole-s [b]
eV_30 + TBMA => TBMA+ + ionization30 + 2 LEE	8.50 x10^{14} L/mole-s [b]
TBMA+ + LEE => TBMA+* + TE + TEformation	1.70 x10^{14} L/mole-s [b]
TBMA+* => TBMA+ + esdecay	2x10^{6} s^{-1} [c]
TBMA + LEE => TBMA* + TE + TEformation	1.70 x10^{14} L/mole-s [b]
TBMA* => TBMA + esdecay	2x10^{6} s^{-1} [c]
TE + TBMA+ => TBMA + TErecombination	4.1x10^{13} L/mole-s [e]
TPS+ + excite => TPS+2 + eV_80 + TPS+_absorb	6.86x10^{12} s^{-1} [a]
eV_80 + TPS+ => TPS+2 + ionization80 + eV_55 + LEE	1.40x10^{15} L/mole-s [b]
eV_55 + TPS+ => TPS+2 + ionization55 + eV_30 + LEE	1.20 x10^{15} L/mole-s [b]
eV_30 + TPS+ => TPS+2 + ionization30 + 2 LEE	8.50 x10^{14} L/mole-s [b]
TPS+ + LEE => TPS+* + TE + TEformation	1.70 x10^{14} L/mole-s [b]
TPS+* => TPS+ + esdecay	2x10^{6} s^{-1} [c]

TPS+2 + LEE => TPS+2* + TE + TEformation	1.70 x10^{14} L/mole-s [b]
TPS+2* => TPS+2 + esdecay	2x10^{6} s^{-1} [c]
TE + TPS+ => TPS0 + TErecombination	4.1x10^{13} L/mole-s [e]
TE + TPS+2 => TPS+ + TErecombination	4.1x10^{13} L/mole-s [e]

[a] Calculated as shown in Tables S3 and S4. The rates are zeroth order in the species excite, it is only present to allow an ionization event to take place
[b] electron impact ionization rate coefficient
[c] mid-range value for non-radiative relaxation from a triplet manifold[2]
[d] electron attachment rate coefficient
[e] recombination rate coefficient

Rate coefficients for electron-cation recombination and electron attachment to form anions are calculated using the following expressions.[3] The second order electron-cation recombination coefficient k_r, in cc/molecule-s, is given by the reduced Debye equation, valid for non-polar, solid media

$$k_r = \frac{4\pi e\mu}{\epsilon} \qquad (1)$$

where e is the elementary charge (esu), μ is the electron mobility and ε is the dielectric constant. Values for mobility and dielectric constant are not available for the specific composition studied in this work, accordingly those for PHS were used as a proxy: μ = 0.114 (cm^2/V-s) and ε = 3 at low frequency. The units for k_r are converted to L/mole-s for this study using Avogadro's number, cm^3 to liters and abvolts to volts, yielding

$$k_r = 1.09 \times 10^{15} \frac{\mu}{\epsilon} \qquad (2)$$

The second order electron-neutral attachment rate coefficient is calculated from the Smoluchowski equation

$$k_a = \frac{4\pi kT \rho_a}{\mu} \qquad (3)$$

where k is Boltzmann's constant in J/K, T is temperature, assumed to be 298K, ρ_a is the electron attachment radius,[4] 1.4x10^{-7}cm, and μ is as defined above. k_a is converted from cm^3/molecule-s to L/mole-s. The value estimated here is consistent with the literature, where rate coefficients in liquid for nondissociative electron attachment are in the range of 4 x 10^{12} L/mol-sec.[5]

The EUV ionization rate coefficients presented in Table S4 are calculated by normalizing the molecular absorption cross sections in Table S3 to the total molecular cross section for the composition, 7.65791x10^{-17} cm^2, and multiplying by an ionization rate coefficient of 4.5x10^{13} s^{-1}. This coefficient is taken from gas phase measurements of nitroaniline ionization using 20-50 eV attosecond pulses.[6] Because each spur results from a single photoexcitation event, having a reasonable order of magnitude for the photoionization rate coefficient is adequate, although more precise numbers for ultrafast ionization in polymers would be very valuable.

Table S3. Calculation of absorption cross sections for elements present in the films

Element	Mass (g)	Absorption cross section cm^2/g [7]	Absorption cross section, cm^2/atom
H	1.008	14900	2.49E-20
C	12.011	29109	5.80E-19
N	14.007	51964	1.21E-18
O	15.999	78944	2.10E-18
F	18.9984	102700	3.24E-18
S	32.06	16105	8.57E-19

Table S4. Calculation of ionization rate coefficients for molecular species

Molecular species	composition	Total ionization cross section per molecule, cm^2	Normalized rate coefficient, s^{-1}
quencher-	$C_8H_4NO_2$	$1.0146x10^{-17}$	$5.96\ x10^{12}$
PAG-	$C_4F_9O_3S$	$3.8625x10^{-17}$	$2.27\ x10^{13}$
PHS	C_8H_8O	$6.9404x10^{-18}$	$4.08\ x10^{12}$
TBMA	$C_8H_{14}O_2$	$9.187\ x10^{-18}$	$5.40\ x10^{12}$
TPS+	$C_{18}H_{15}S$	$1.168\ x10^{-17}$	$6.86\ x10^{12}$

In the model, the molecular components, marker species (Table S1) and the initial EUV photon are constrained to stay in their initial compartments, however all electron species are allowed to diffuse between compartments. High energy electrons (80, 55 and 30 eV) are assumed to travel only outward from the first compartment, ie do not backscatter, while LEE and TE are allowed to diffuse in both directions. Jump diffusion coefficients, shown in Table S5, are estimated from the electron kinetic energies using the expression[8]

$$D = < \frac{1}{\tau} > < r^2 > \quad (4)$$

where τ (s) is the time required for an electron to travel a distance $r = 1$ nm, approximating the mean free path between scattering events of 0.79 nm as determined for poly(methylmethacrylate).[9]

Table S5. Diffusion coefficients for electrons[a]

Electron energy eV	Electron velocity m/s	time to move 1 nm, s	Diffusion coefficient nm^2/s
80	$5.305x10^6$	$1.885x10^{-16}$	$5.305x10^{15}$
55	$4.399x10^6$	$2.273x10^{-16}$	$4.399x10^{15}$
30	$3.249x10^6$	$3.078x10^{-16}$	$3.249x10^{15}$
5	$1.326x10^6$	$7.540x10^{-16}$	$1.326x10^{15}$
0.1	$1.876x10^5$	$5.332x10^{-15}$	$1.876x10^{14}$

[a] 80, 55 and 30 eV electrons are assumed to only diffuse away from the first compartment where photoabsorption occurs. 5 eV (LEE) and 0.1eV (TE) electrons diffuse in both directions

Section S3. Theoretical estimates of ionization energies

To calculate vertical electron affinities and ionization energies, structures previously optimized with B97M-V[10]/def2-SVPD[11] and C-PCM[12] with a dielectric of 3.0 were reoptimized with ωB97M-V[13]/def2-TZVPD[11] and C-PCM with a dielectric of 3.0. Single point calculations were then performed with charge increased or decreased by 1 to obtain the change in energy associated with instantaneous removal or addition of an electron. All calculations were performed with Q-Chem 6.1[14] via workflows written in Atomate[15] leveraging on-the-fly error correction as implemented in Custodian[16] and Q-Chem default parameters implemented in Pymatgen.[17] The use of vertical rather than adiabatic values was justified by the rigid polymer environment, which inhibits rapid structural relaxation/rearrangement due to electron attachment/detachment. Table S6 presents ionization free energies and electron affinities calculated for the present study. Gas phase values for the electron affinity of methyl methacrylate (MMA) are available for comparison and show that it does not form a stable anion in agreement with the calculations.[18] Values for PHS have not been previously reported.

Table S6. Energetics for electron detachment (ionization) and attachment (electron affinity)

**Electron detachment**		
**Species**	**ΔG (eV)**	
$TPS^+ \rightarrow TPS^{2+}$	9.31	
$PHS \rightarrow PHS^+$	6.63	
$TBMA \rightarrow TBMA^+$	8.00	
$quencher^- \rightarrow quencher$	5.49	
$PAG^- \rightarrow PAG$	6.90	
**Electron attachment (EA)**		
**Species**	**Vertical EA**	**Adiabatic EA**
$TPS^+ \rightarrow TPS$	2.37	2.81
$PHS \rightarrow PHS^-$	-0.01	0.18
$TBMA \rightarrow TBMA^-$	-0.85	0.14
$MMA \rightarrow MMA^-$	0.35	-0.19

Section S4. Density measurement by X-ray reflectivity

The measurements of EUV absorption of a 38 nm thick 40% PHS 60% TBMA film were performed at the Advanced Light Source (ALS) beamline 6.3.2 at the Lawrence Berkeley National Laboratory.[19] Reflectivity measurements at 13.5 nm were conducted using a 200 mm^{-1} grating, a Be filter, and a photodiode detector. The components of complex refractive index $\tilde{n}$ are given as

$$\tilde{n} = 1 - \delta + i\beta = 1 - \frac{r_e}{2\pi}\lambda^2 \sum_i n_i f_i(0) \quad (5)$$

where δ is the dispersive component and β is the absorptive component of the refractive index of polymer film. The imaginary part β of the refractive index is obtained by fitting experimental data.

The δ and β are related to the sum of atomic scattering factors of the elements constitute the molecule,[20] r_e is the classical electron radius, λ is the x-ray wavelength, n_i is the number of atoms (or molecules) per unit volume and $f_i(0)= f_1-if_2$ is the atomic scattering factor. Tables of values for f_1 and f_2 have been published.[7] The mass density ρ (g/cm^3) is obtained from

$$n_i = \frac{\rho N_A}{MW} \tag{6}$$

where N_A is the Avogadro's number, and MW is the molecular weight of the polymer.

The polymer film density is determined to be 1.075 g/cm^3. The extracted values of δ and β from the reflectivity measurement are $0.0221 \pm 2.51\times10^{-5}$ and $0.0042 \pm 4.78\times10^{-6}$, respectively, and the error in ρ is that for δ. The uncertainties derive from the assumption that the film is homogeneous, its thickness is precise, and that the 40:60 composition of PHS:TBMA is exact. Any inhomogeneities will affect the density value, and for the present work only one film was examined. Accordingly we use the uncertainty reported in a much more extensive study of ultrathin resist polymer densities in this work.[21]

Section S5. Additional simulation results

The Kinetiscope[22] simulations provide complete time histories of all component concentrations and diffusion steps as a function of location. These results are exported as an xmf file and analyzed using python scripts and spreadsheets to extract specific data sets for analysis and visualization.

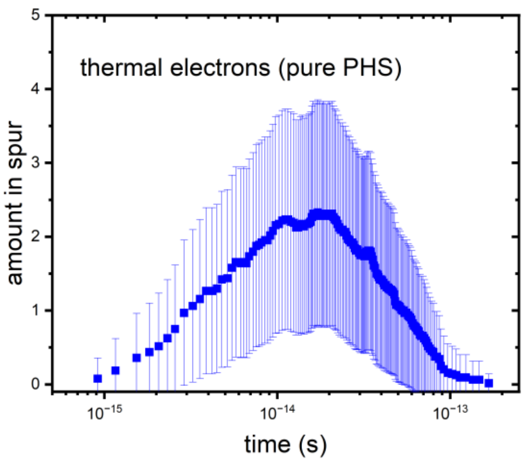

Figure S2. Thermal electron time history within a 1-D 62-compartment array for pure PHS. The mean and standard deviation from 64 instances are shown.

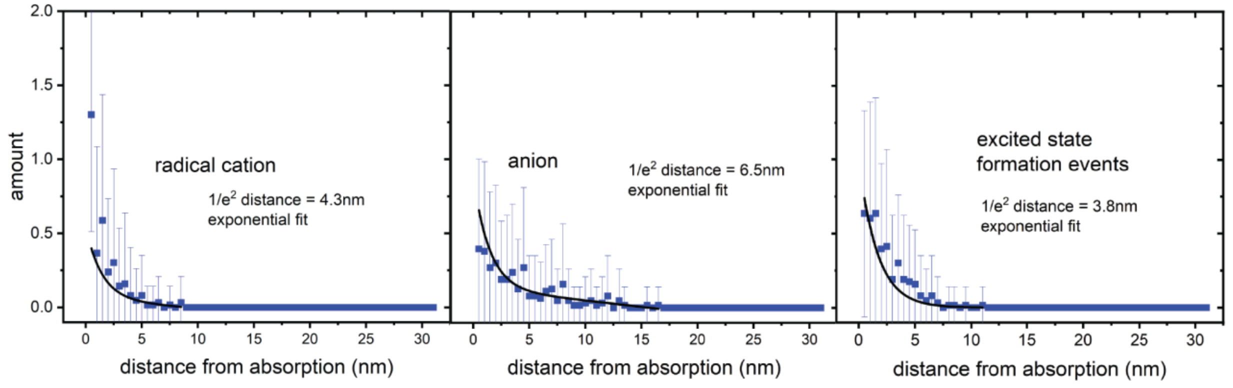

Figure S3. Fits for data in Figure 3, main paper. Terminal spatial distribution of primary radiolysis products in PHS, numbers per $5.1 nm^3$ compartment as a function of distance from the first compartment where EUV absorption occurs. (a) PHS radical cations, (b) PHS anions, (c) location of excited state formation events occurring throughout the simulation.

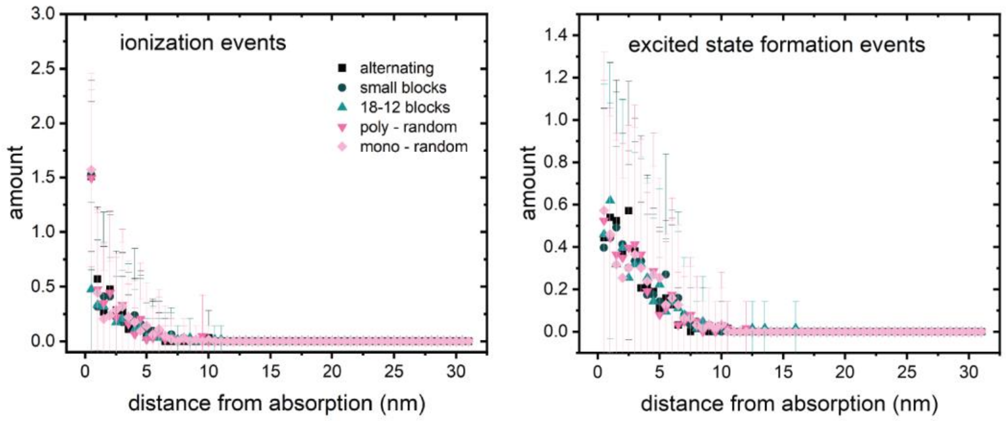

Figure S4 Location of ionization and electronic excitation events in the photoresist simulations, amounts per $5.15 nm^3$ compartment in the 1-D array.

Table S7. Total numbers of primary radiolysis products remaining in spur at end of simulation, per photon absorbed[a]

Film	Radical cations	Neutral radicals	Radical anions
Resist, monodisperse random	3.0 ± 1.7	3.3 ± 1.8	0.7 ± 0.9
Resist, polydisperse random	3.0 ± 1.7	3.3 ± 1.8	0.8 ± 0.9
Resist, defined sequence alternating	3.0 ± 1.7	3.4 ± 1.8	0.7 ± 0.8
Resist, defined sequence small blocks	3.0 ± 1.7	3.4 ± 1.8	0.7 ± 0.8
Resist, defined sequence 8-12 blocks	3.1 ± 1.8	3.2 ± 1.8	0.8 ± 0.9
Poly(hydroxystyrene)	3.4 ± 1.8	--	3.4 ± 1.8

a. Determined from numbers of radical cations (PHS^+, $TBMA^+$, TPS^{+2}), radical anions (PHS^-), neutral radicals (PAG^0, $quencher^0$, TPS^0) and standard error propagation for standard deviations of each quantity.

References

(1) (a) *Polyscope*; https://github.com/hnsbrg/PolyScope, 2024. (accessed August 27, 2025). (b) Hinsberg, W. D.; Houle, F. A. Comparison of the spatial statistics of random and defined-sequence photoresist films. *J Micro-Nanopattern* **2024**, *23* (4), Artn 044601. DOI: 10.1117/1.Jmm.23.4.044601.
(2) Elsayed, M. A. Triplet State - Its Radiative and Nonradiative Properties. *Accounts Chem Res* **1968**, *1* (1), 8-16. DOI: DOI 10.1021/ar50001a002.
(3) Kim, Y. J.; Kim, J.; Kim, Y. S.; Lee, J. K. TiO-poly(4-vinylphenol) nanocomposite dielectrics for organic thin film transistors. *Org Electron* **2014**, *15* (2), 640-640. DOI: 10.1016/j.orgel.2013.12.005.
(4) Orient, O. J.; Chutjian, A. Comparison of Calculated and Experimental Thermal Attachment Rate Constants for Sf6 in the Temperature-Range 200-600-K. *Phys Rev A* **1986**, *34* (3), 1841-1846. DOI: 10.1103/PhysRevA.34.1841.
(5) Christophorou, L. G. The dependence of the thermal electron attachment rate constant in gases and liquids on the energy position of the electron attaching state. *Z Phys Chem* **1996**, *195*, 195-215. DOI 10.1524/zpch.1996.195.Part_1_2.195.
(6) Vismarra, F.; Fernández-Villoria, F.; Mocci, D.; González-Vázquez, J.; Wu, Y. X.; Colaizzi, L.; Holzmeier, F.; Delgado, J.; Santos, J.; Bañares, L.; et al. Few-femtosecond electron transfer dynamics in photoionized donor-acceptor molecules. *Nat Chem* **2024**, *16* (12). DOI: 10.1038/s41557-024-01620-y.
(7) Henke, B. L.; Gullikson, E. M.; Davis, J. C. X-Ray Interactions - Photoabsorption, Scattering, Transmission and Reflection at E=50-30,000 Ev, Z=1-92. *Atom Data Nucl Data* **1993**, *55* (2), 349-349.
(8) Bisquert, J. Interpretation of electron diffusion coefficient in organic and inorganic semiconductors with broad distributions of states. *Phys Chem Chem Phys* **2008**, *10* (22), 3175-3194. DOI: 10.1039/b719943k.
(9) Tanuma, S.; Powell, C. J.; Penn, D. R. Calculations of Electron Inelastic Mean Free Paths .5. Data for 14 Organic-Compounds over the 50-2000 Ev Range. *Surf Interface Anal* **1994**, *21* (3), 165-176. DOI: 10.1002/sia.740210302.
(10) Mardirossian, N.; Head-Gordon, M. Mapping the genome of meta-generalized gradient approximation density functionals: The search for B97M-V. *Journal of Chemical Physics* **2015**, *142* (7), Artn 074111. DOI: 10.1063/1.4907719.
(11) (a) Rappoport, D.; Furche, F. Property-optimized Gaussian basis sets for molecular response calculations. *Journal of Chemical Physics* **2010**, *133* (13), Artn 134105. DOI: 10.1063/1.3484283. (b) Hellweg, A.; Rappoport, D. Development of new auxiliary basis functions of the Karlsruhe segmented contracted basis sets including diffuse basis functions (def2-SVPD, def2-TZVPPD, and def2-QVPPD) for RI-MP2 and RI-CC calculations. *Phys Chem Chem Phys* **2015**, *17* (2), 1010-1017. DOI: 10.1039/c4cp04286g.
(12) (a) Barone, V.; Cossi, M. Quantum calculation of molecular energies and energy gradients in solution by a conductor solvent model. *J Phys Chem A* **1998**, *102* (11), 1995-2001. DOI 10.1021/jp9716997. (b) Cossi, M.; Rega, N.; Scalmani, G.; Barone, V. Energies, structures, and electronic properties of molecules in solution with the C-PCM solvation model. *J Comput Chem* **2003**, *24* (6), 669-681. DOI: 10.1002/jcc.10189.
(13) Mardirossian, N.; Head-Gordon, M. ωB97M-V: A combinatorially optimized, range-separated hybrid, meta-GGA density functional with VV10 nonlocal correlation. *Journal of Chemical Physics* **2016**, *144* (21), Artn 214110. DOI: 10.1063/1.4952647.

(14) Epifanovsky, E.; Gilbert, A. T. B.; Feng, X. T.; Lee, J.; Mao, Y. Z.; Mardirossian, N.; Pokhilko, P.; White, A. F.; Coons, M. P.; Dempwolff, A. L.; et al. Software for the frontiers of quantum chemistry: An overview of developments in the Q-Chem 5 package. *Journal of Chemical Physics* **2021**, *155* (8), Artn 084801. DOI: 10.1063/5.0055522.
(15) Mathew, K.; Montoya, J. H.; Faghaninia, A.; Dwarakanath, S.; Aykol, M.; Tang, H. M.; Chu, I. H.; Smidt, T.; Bocklund, B.; Horton, M.; et al. Atomate: A high-level interface to generate, execute, and analyze computational materials science workflows. *Comp Mater Sci* **2017**, *139*, 140-152. DOI: 10.1016/j.commatsci.2017.07.030.
(16) Blau, S. M.; Spotte-Smith, E.; Wood, B.; Dwaraknath, S.; Persson, K. A. Accurate, Automated Density Functional Theory for Complex Molecules Using On-the-fly Error Correction. *ChemRxiv* **2020**. DOI: 10.26434/chemrxiv.13076030.v1.
(17) Ong, S. P.; Richards, W. D.; Jain, A.; Hautier, G.; Kocher, M.; Cholia, S.; Gunter, D.; Chevrier, V. L.; Persson, K. A.; Ceder, G. Python Materials Genomics (pymatgen): A robust, open-source python library for materials analysis. *Comp Mater Sci* **2013**, *68*, 314-319. DOI: 10.1016/j.commatsci.2012.10.028.
(18) Luxford, T. F. M.; Fedor, J.; Kocisek, J. Electron Energy Loss Processes in Methyl Methacrylate: Excitation and Bond Breaking. *Acs Biomater Sci Eng* **2023**, *127* (12), 2731-2741. DOI: 10.1021/acs.jpca.2c09077.
(19) Kostko, O.; McAfee, T. R.; Ma, J.; Blackwell, J. M.; Naulleau, P. Experimental characterization of model extreme ultraviolet resist materials. *J Micro-Nanopattern* **2024**, *23* (1), Artn 014602. DOI: 10.1117/1.Jmm.23.1.014602.
(20) Yan, H. P.; Wang, C.; McCarn, A. R.; Ade, H. Accurate and Facile Determination of the Index of Refraction of Organic Thin Films Near the Carbon 1 Absorption Edge. *Phys Rev Lett* **2013**, *110* (17), ARTN 177401. DOI: 10.1103/PhysRevLett.110.177401.
(21) Goldfarb, D. L.; Lin, Q. H.; Angelopoulos, M.; Soles, C. L.; Lin, E. K.; Wu, W. Characterization of thin and ultrathin polymer and resist films. *Advances in Resist Technology and Processing XVIII, Pts 1 and 2* **2001**, *4345*, 335-343. DOI: 10.1117/12.436863.
(22) Hinsberg, W. D.; Houle, F. A. *Kinetiscope, available at www.hinsberg.net/kinetiscope*. 2025. (accessed August 27, 2025).
